# The Role of Obsessive Compulsive Traits in Fibromyalgia: Is Pain-Related Obsessive Ideation Involved in Pathogenesis?

**DOI:** 10.3390/medicina60071027

**Published:** 2024-06-23

**Authors:** Bat-El Lugassy-Galper, Mor Amital, Howard Amital, Dan Buskila, Daniela Amital

**Affiliations:** 1 Department of Medicine B & The Center for Autoimmune Diseases, Sheba Medical Center, Tel-HashomerRamat-Gan 5262100, Israel; batellug@gmail.com; 2 Faculty of Medical & Health Sciences, Tel Aviv University, Tel Aviv 6997801, Israel; 3 The Adelson School of Medicine, Ariel University, Ariel 4077625, Israel; m.moramital@gmail.com; 4 Faculty of Health Sciences, Ben Gurion University of the Negev, Beer Sheva 8410501, Israel; dbuskila@bgu.ac.il (D.B.); danielaam@bmc.gov.il (D.A.); 5 Division of Psychiatry, Barzilai Medical Center, Ben Gurion University of the Negev, Ashkelon 7830604, Israel

**Keywords:** fibromyalgia, obsessive compulsive symptoms (OCS), obsessive compulsive disorder (OCD), pain, disability

## Abstract

*Background and Objectives*: Fibromyalgia syndrome (FMS) is defined as a chronic pain syndrome that is characterized by widespread pain, tenderness, and diffuse stiffness. In addition, neuropsychological symptoms such as fatigue, sleep disorders, poor mood, cognitive impairment, and headaches are often reported. Many reports have addressed the coexistence of affective disorders and anxiety with FMS, yet few have focused on its association with obsessive compulsive disorder (OCD). We investigated the occurrence of classical patterns of OCD in participants with FMS and assessed their effect on pain perception and functional impairment. *Material and Methods*: The research population included 37 patients diagnosed with FMS, treated at the Rheumatology Clinic in the Sheba Medical Center, Tel-Hashomer, Israel. We used validated questionnaires including a demographic questionnaire, a questionnaire on average and maximal pain intensity, the Eysenck Personality Questionnaire-Revised (EPQ-R), the Perceived Stress Scale, the Pain Catastrophizing Scale, the Pain Obsessive questionnaire, and the Yale–Brown Obsessive Compulsive Scale (Y-BOCS). *Results*: Patients with FMS were found to have intrusive and obsessive thoughts regarding pain for several hours every day, causing a high degree of anxiety and high levels of pain, catastrophizing, and magnification, leading to helplessness and functional impairment. In total, 27% of the patients reported severe malfunction due to pain and pain ideation, and 49% demonstrated mild obsessive compulsive symptoms that were strongly correlated with pain intensity and functional impairment. *Conclusions*: Obsessive compulsive thinking patterns contribute to pain magnification and to the cognitive aspects of fibromyalgia syndrome.

## 1. Introduction

Fibromyalgia syndrome (FMS) is a common cause of chronic widespread pain, characterized by tenderness, diffuse stiffness, as well as fatigue, unrefreshing sleep, dysphoria, cognitive disorders, and headaches. FMS is the most common cause of diffuse musculoskeletal pain in women aged 20–55 [1]. It is more common in women compared to men, by a ratio of 7–9:1, with a prevalence ranging from 2 to 4%, which increases with age. FMS has also been described in children [2].

The diagnosis of FMS, similar to other somatic functional disorders, is controversial due to discrepancies between reported symptoms and the lack of objective findings. Consequently, the etiology of the syndrome has been considered by many medical care providers to be primarily of psychological and psychosomatic origin. However, it has been demonstrated that FMS develops due to various genetic, epigenetic, hereditary, and environmental causes, as well as neurological mechanisms, which all lead to enhanced pain perception by central pain sensitization [3]. Nevertheless, there seems to be a role for distress and exposure to traumatic events, which facilitates the emergence of FMS. Other potential triggers include infectious diseases and vaccinations, which may also precipitate a fibromyalgic process [4].

Fibromyalgia often coexists with other diseases such as hypertension (34.8%), metabolic disorders (17.8%), atherosclerosis (16.3%) and diabetes mellitus (14.8%), as well as depression, anxiety, and posttraumatic stress disorder (30–50%) [5,6,7,8]. The association between fibromyalgia and obsessive compulsive disorder (OCD) has not been thoroughly studied. OCD is characterized by intrusive repetitive thought patterns, impulses, or obsessions that cause anxiety and stress, resulting in compulsive behaviors that the individual is determined to follow. These obsessive compulsive symptoms are time-consuming, occupying at least one hour a day, and may cause major distress or functional impairment [9].

The prevalence of the disorder is estimated to occur in 1–2% of the general population [10]. However, several studies demonstrated a higher rate of OCD among patients with fibromyalgia, i.e., 4–6 times higher [10,11]. Moreover, it has been demonstrated that obsessive compulsive personality disorder is six times more common among patients with fibromyalgia [12] than among subjects without fibromyalgia [11]. In addition, women with fibromyalgia are five times more likely to develop OCD during their lifetime [13].

Obsessive thought patterns include rumination, repetitive negative thoughts leading to distress, a sense of helplessness [14,15,16], and increased anxiety. Additionally, pain ruminations increase emotional distress and may contribute to higher perceived pain intensity [16], leading to pain catastrophizing, i.e., the characterization of pain as horrible and unbearable [17]. Since 1989, several studies indicated the importance of pain catastrophizing in the experience of chronic pain [18,19,20,21], and it may be responsible alone for the transition of acute pain to the form of chronic pain [22].

In patients with fibromyalgia, it has been shown that pain catastrophizing magnifies pain perception through enhanced attention to painful stimuli and heightened emotional responses to pain by activation of brain structures which mediate pain sensation [17]. Pain catastrophizing is also associated with higher disability rates and may be a more significant negative functional impairment predictor than organic symptoms [22].

In this study, we investigated obsessive thought patterns regarding pain among patients with fibromyalgia. Our hypothesis was that obsessive thought patterns cause pain catastrophizing, leading to pain magnification that increases anxiety, distress, and functional impairment. In addition, we assumed that there is a positive correlation between obsessive compulsive symptoms and reported pain intensity, leading to increased functional impairment.

## 2. Material and Methods

Our study included 37 patients diagnosed with fibromyalgia, treated at the Rheumatology Clinic of Sheba Medical Center, Tel-Hashomer, Israel. The study was approved by the Sheba Helsinki committee (1580-14-SMC, 9.2014). The Helsinki committee accepted the completion of the above-mentioned questionnaires by telephone conversation as an acceptance of consent. All patients approved their participation in the study prior to answering the questions presented to them.

The inclusion criteria were women and men above the age of 18 who were primarily diagnosed with fibromyalgia. Exclusion criteria were the following: pregnant women, breast feeding women, neoplastic diseases (aside from basal cell carcinoma), other rheumatologic conditions, inflammatory diseases, and any comorbidities which may cause severe disability. The sample size was not precalculated. The questionnaires were completed by a telephone call by M. Amital following the clinical encounter.

The research design was based on the following questionnaires:

1. **Demographic questionnaire**—including age, sex, marital status, education level, duration of disease, medications, comorbidities, past and/or recent mental treatment.
2. **Mean and maximum pain intensity questionnaire**—using a visual analog score (VAS) scale, regarding the last week.
3. **Eysenck Personality Questionnaire—Revised (EPQ-R)** [23]—A validated questionnaire that evaluates personality traits in four dimensions: extraversion, neuroticism, psychoticism, and social desirability. In this study, we focused on the neuroticism dimension alone in order to evaluate obsessive traits, anxiety, depression, low self-esteem, guilt thoughts, etc.
4. **Perceived Stress Scale questionnaire (PSS14)** [24]—A validated questionnaire comprised of 14 items regarding feelings and thoughts during the last month, for measuring the perception of stress. Total scores ranging from 0 (stress absence) to 56 (maximal stress) are divided into 3 groups: low stress (score ≤ 18), moderate stress (18 < score ≤ 37), and high stress (score > 38).
5. **Pain Catastrophizing Scale questionnaire** [25]—A validated questionnaire. Pain catastrophizing is characterized by the tendency to magnify pain stimuli by ruminating on pain thoughts, causing helplessness and difficulty to control and prevent these pain thoughts. Therefore, pain catastrophizing affects pain perception and consequently impacts quality of life. The questionnaire quantifies one’s pain experience in three aspects: pain magnification, pain rumination, and helplessness. Raw scores are converted into percentiles, where the 75th percentile reflects high pain catastrophizing.
6. **Pain Obsessive Thoughts questionnaire**—Quantifies the time period of thoughts about pain, the anxiety caused by these thoughts, and the ability to control them. Moreover, the questionnaire quantifies the impact on social and occupational functions as a result of repetitive pain thoughts. This is not a validated score.
7. **Obsessive Compulsive Test—Yale Brown OCD Scale (Y-BOCS)** [26]—A validated questionnaire which enables evaluation of obsessive compulsive disorder severity. The scores are divided into 5 groups: subclinical disorder (score ≤ 7), mild disorder (8–15), moderate disorder (16–23), severe disorder (24–32), and extreme disorder (32–40). In this study, patients were divided into 2 groups: fibromyalgia patients with obsessive compulsive symptoms (OCS), (Y-BOCS score ≥ 1) and fibromyalgia patients without obsessive compulsive symptoms (Y-BOCS score = 0).

The minimal calculating sample size was 36 patients, enabling a correlation of 0.45 with 95% significance.

## 3. Statistical Analysis

Based on the questionnaires, we built a database and analyzed it using SPSS version 25. Spearman’s correlation coefficient was used to evaluate the association between pain intensity and all parameters from other questionnaires. The association between obsessive compulsive symptoms and the other parameters from the questionnaires was analyzed in two manners: qualitative variables were analyzed using the Fisher exact test and continuous variables were analyzed using a *t*-test. Significance was set at *p* ≤ 0.05. In addition, we used multivariate linear regression in order to find the parameters that independently affect the pain intensity reported by patients. Moreover, we conducted a multivariate logistic regression to compare patients with and without OCD traits.

Our independent variables are continuous measures rather than categorical variables, therefore there is no reference group in the traditional sense. The statistical significance (*p*-value) pertains to the hypothesis that the coefficient for the predictor is different from zero.

## 4. Results

The demographic data of the participants are presented in Table 1. As expected, 95% were females, with a mean age of 54 years, compared to 5% men with a mean age of 27 years. The disease duration was longer in the female group (mean of 6.3 years compared to 3.75 years in the male group). More than half of the patients reported other comorbidities, all of them female. The most common comorbidities were hypertension (38%), osteoporosis (19%), and diabetes mellitus (14%), asthma (10%) and hypothyroidism (10%), similar to rates reported previously [27].

Regarding OCS, the mean Y-BOCS score was 6.6 ± 9.0, which reflects subclinical obsessive compulsive symptoms among the research participants (*n* = 37). Based on Y-BOCS scores, patients were divided into two groups: fibromyalgia with OCS (Y-BOCS ≥ 1) and fibromyalgia without OCS. Fifty-one percent of the research participants had obsessive compulsive symptoms according to this criterion (19/37). In the group of fibromyalgia with OCS, the mean Y-BOCS score was 13.4 ± 6.7, with subclinical OCD in four participants (21%), mild OCD in eleven (58%), moderate OCD in two (11%), and severe OCD in two (11%).

**Figure 1:**
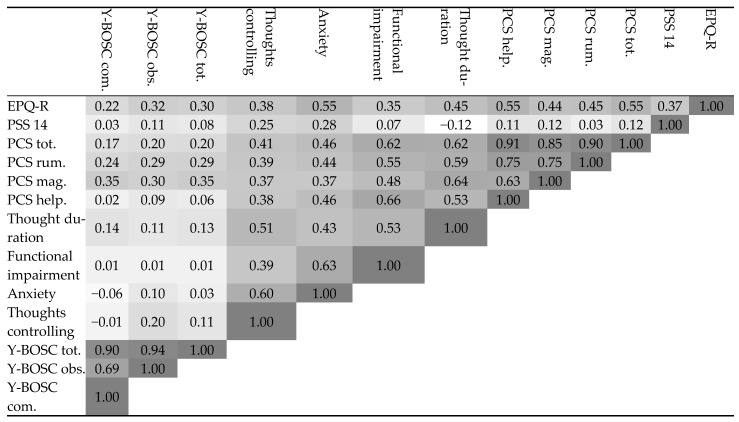
Pearson correlations summary in the group of fibromyalgia and obsessive compulsive symptoms (OCS). The darker the cell color, the more statistically significant it is (*p* < 0.05). Cell darkness indicates higher significance. Abbreviations: PSS14—Perceived Stress Scale questionnaire, PCS—Pain Catastrophizing Scale, PCS.tot = PCS questionnaire total score, PCS.rum = PCS rumination component score, PCS.mag = PCS magnification component score, PCS.help = PCS helplessness component score, Y.BOSC.TOT = Y.BOSC questionnaire total score for evaluating OCD severity, Y.BOSC.obs = Y.BOSC obsession component, Y.BOSC.com—Y.BOSC compulsion component.

Regarding the obsessive component, individuals with fibromyalgia and OCS received a mean score of 8 ± 4, reflecting mild obsession. The most frequent obsessive traits were somatic obsessions, checking obsessions, religious obsessions, fear of losing things, symmetry and ordering, and contamination obsessions (Table 2). In addition, in the group of individuals with fibromyalgia and OCS, a strong positive correlation was found between obsessive compulsive symptoms (OCS) and functional impairment (R = 0.6, *p* = 0.009) (Figure 1).

In the compulsive component, the fibromyalgia with OCS group received a mean score of 5.4 ± 4.2, reflecting subclinical compulsions. The most frequent were washing and cleaning (31%), rechecking (17%), and ordering and arranging (17%).

The mean pain score reported was similar in men and women, 3.5 ± 0.7 and 4 ± 1.2, respectively, reflecting high pain intensity. Stratification by gender, education level, and mental therapy was statistically insignificant, but a tendency to report higher pain intensity was observed in women and in participants with a lower education level. In addition, a tendency to report lower pain intensity was observed in patients who had received mental therapy of any type in the past.

Average and maximal pain differences were not significantly higher between fibromyalgia patients with OCS and without, but a tendency to report a higher pain intensity was observed in the OCS group (average pain of 4.1 vs. 3.8; maximal pain 5 vs. 4.6). However, in the OCS group a positive correlation with statistical significance was found between average and maximal pain and functional impairment (Figure 1).

The EPQ-R questionnaire analysis indicated a mean score of 5.9 ± 3.12, reflecting moderate neuroticism among the study population (*n* = 37); 30% displayed high neuroticism (11/37), 38% moderate neuroticism (14/37), and 32% mild neuroticism (12/37). Neuroticism was twice as high in the OCS group compared to patients without OCS, a difference with no statistical significance (a median score of 7 versus 4, respectively).

Measuring stress levels in the study population (PSS-14 questionnaire) demonstrated that 97% (36/37) had a moderate stress level with a mean score of 25.7 ± 4.2. Higher stress levels were demonstrated in the OCS group, a difference that was not significant.

A high pain catastrophizing score was observed in the study population (*n* = 37) by using the Pain Catastrophizing Scale (PCS) questionnaire, reaching the 75.2 percentile, based on pain rumination, magnification, and helplessness (Table 3). Higher scores were obtained in all questionnaire parameters in the FMS with OCS group compared to the fibromyalgia without OCS (*p* < 0.05) group, as follows according to their percentiles: PSC rumination 78 vs. 65, PCS magnification 75 vs. 59, PCS helplessness 83 vs. 79 and total PCS 80 vs. 70.

Moreover, in the OCS group, a statistically significant positive correlation was found between pain catastrophizing (rumination and helplessness components) and functional impairment (R = 0.48, R = 0.7, and R = 0.56, respectively) (Figure 1). Seventy five percent of the participants had thoughts regarding pain for a moderate-to-long duration (1–3 h to more than 8 h every day), 78.3% (29/37) reported moderate-to-high functional impairment due to these thoughts, and 25% reported a lack of functioning.

Moderate-to-high anxiety was reported by 67.5% of the participants and was attributed to pain thoughts; 62% of the patients reported difficulty controlling these pain thoughts. Regarding pain thought duration, functional impairment, anxiety level, and the ability to control pain thoughts, no significant statistical difference was found between fibromyalgia patients with or without OCS (*p* > 0.05). However, applying multivariate linear regression using pain intensity as the dependent variable demonstrated that pain catastrophizing, including rumination, magnification, and helplessness, affected pain intensity. These parameters were significantly influential among the subjects with fibromyalgia and OCS compared to those without OCS (Table 4). Moreover, increased pain intensity in the fibromyalgia with OCS group explained functional impairment and increased anxiety significantly (Table 5).

In addition, we detected a significant positive correlation between pain catastrophizing and obsessive pain thought duration (R = 0.62). In addition, a significant positive correlation was detected between pain catastrophizing and functional impairment (R = 0.62) (Figure 1) and between obsessive pain thought duration and anxiety (R = 0.43).

## 5. Discussion

Fibromyalgia is characterized by musculoskeletal pain and involves a reduced pain threshold, hyperalgesia, and allodynia [28]. Fatigue, sleep disorders, functional impairment, cognitive disorders, mood changes, stiffness, urinary disorders, and gastrointestinal tract symptoms are also common [29].

The association between fibromyalgia and other diseases, including psychiatric disorders, has been extensively studied. Accompanying psychiatric and rheumatic disorders have been shown to be associated with increased disability, mortality, and impaired quality of life [30]. The most common investigated psychiatric comorbidities of fibromyalgia are major depressive and anxiety disorders [31,32]. However, the association between fibromyalgia and obsessive compulsive disorder (OCD), which may affect pain perception and overall function, has yet to be thoroughly investigated.

In this study, we examined the linkage between fibromyalgia and OCS as well as the association between reported pain intensity as an outcome of obsessive pain ideation and its’ impact on function and disability. To the best of our knowledge, no studies have investigated this previously.

The data analysis supports the existence of obsessive pain thoughts in patients with fibromyalgia, which cause high stress levels and high pain catastrophizing, leading to increased anxiety elicited by a difficulty to control these thoughts. Obsessive pain thoughts were shown to generate a cascade of functional impairment and disability. The patients were recruited for the study based on their arrival at the clinic and their consent. The low number of male participants reflects their lower representation in the fibromyalgia patient population. We chose to include these participants in the study to ensure that our analysis accurately represents the true distribution seen in our daily clinical practice. We understand that this might have generated certain biases due to the difference in the clinical demographics between the sexes.

The results of this study raise the question of whether there is an increased prevalence of OCD among patients with fibromyalgia. Previous research has shown an increased prevalence of obsessive compulsive personality disorder, but findings regarding OCD have been inconclusive [30,33]. A limited number of studies have indicated an approximately fivefold-increased lifetime risk for OCD and post-traumatic stress disorder among patients with fibromyalgia [13,34,35]. Furthermore, these studies have demonstrated a higher current comorbidity of OCD among fibromyalgia patients.

Understanding whether there is an increased incidence of OCD in fibromyalgia may be important and beneficial in the therapeutic approach, since OCD is a common diagnosis estimated to be the fourth most prevalent lifetime psychiatric disorder [36]. Secondly, both OCD and subclinical OCD patients suffer from impaired quality of life [37], and OCD patients have selective attention to pain-related stimuli [38]. Hezel and colleagues [39] found that patients with severe OCD are often willing to endure significant physical pain as a distraction from emotional stress and low self-esteem. Consequently, the absence of an OCD diagnosis and appropriate treatment may contribute to a vicious cycle of obsessive pain thoughts. This can lead to an intensified pain experience and pain catastrophizing, which, in turn, increases anxiety and functional impairment.

According to the current research results, we assume that OCD among patients with fibromyalgia is underdiagnosed, and therefore, screening for OCD may be useful for effective, integrated and targeted treatment. We believe that pain obsessions may be a contributing factor to fibromyalgia severity.

The limitations of the current study should be mentioned. These include the relatively small sample size, the fact that the study was conducted in a university hospital setting, and the fact that the severity of fibromyalgia was not evaluated by a validated questionnaire such as the FIQ but rather was based on a self-reported functional impairment, assuming that disease severity is directly linked to functional impairment. In addition, the focus of the study was on aspects of obsessive compulsive behavior. We did not plan to explore the impact of different therapies on these symptoms, particularly given the low adherence and high discontinuation rates associated with medication. In addition, it should be mentioned that none of the patients who were offered the opportunity to take part in the study refused, so there were no selection biases in the study.

## 6. Conclusions

In conclusion, the present study demonstrated that a high proportion of patients with fibromyalgia experience prolonged obsessive pain thoughts. These thoughts are associated with pain catastrophizing and magnification, which can lead to high anxiety levels and consequent functional impairment. Screening for OCD among fibromyalgia patients and using combined therapy may assist in coping with the disease and improve functional outcomes.

## Figures and Tables

**Table 1:** Demographic parameters of the research participants.

***n*** **= 37**		
**Mean age**		52.81 (14.65)
**Mean disease duration (years)**		6.34 (6.49)
**Gender (%)**	Female	35 (94.6)
	Male	2 (5.4)
**Marital status (%)**	Married	20 (54.1)
	Divorced	8 (21.6)
	Single	7 (18.9)
	Widowed	2 (5.4)
**Education level**	High school	16 (43.2)
	B.A.	16 (43.2)
	M.A.	5 (13.5)
**Comorbidities (%)**	Yes	21 (56.8)
	No	16 (43.2)
**Medication therapy (%)**	None	6 (16.2)
	Single drug	23 (62.2)
	Two drugs or more	8 (21.6)
**Cannabis use (%)**	Yes	6 (16.2)
	No	31 (83.8)
**Mental care treatment (%)**	Yes	16 (43.2)
	No	21 (56.8)

**Table 2:** Obsession types among FMS participants.

Obsession	% of Participants
Need to know and remember	40
**Somatic obsessions**	
Disease-related concerns	40
Excessive concern for part of the body or about external appearance	20
Religious	31
Fear of losing things	30
Symmetry and accuracy	23
**Infectious obsessions**	
Fear of dirt and bacteria	20
Worry about getting infected	20
Worry that others will start spreading infection	14
Concern/disgust with body secretions	11
Exaggerated concern for environmental pollution	11
Concern about sticky materials/residues	11
Excessive concern for household items	9
Excessive worry about smells	6
No concern about the consequences of infection beyond how we feel	6
**Aggressive obsessions**	
Fear of being responsible for something terrible that will happen	20
Fear of hurting others	17
Fear of doing something embarrassing	14
Scary and violent fictions and images	9
Fear of expressions of obscenity and insults	9
Fear of acting on unwanted urges	9
Fear of hurting myself	6
Fear of hurting others as a result of carelessness	6
Fear of stealing things	3
**Other obsessions**	
Savings, storage, and collection obsessions	11
Fear of saying something	11
Fear of saying the wrong thing	11
Obsessions associated with strange noises and sounds	9
Superstitious fears	9
Intrusive imagery	6
Colors with special meaning	6
Sexual obsessions	0

**Table 3:** Pain Catastrophizing Scale (PCS) questionnaire results, including rumination, magnification, helplessness, and total score among the research group (*n* = 37). SD = standard deviation.

	Raw Score	Percentile	SD
PCS Rumination	10.2	71	3.85
PCS Magnification	8.5	67	2.97
PCS Helplessness	14.3	81.2	4.73
PCS Total	33	75.2	10.53

**Table 4:** Linear regression—Pain Catastrophizing Scale questionnaire (PCS). Dependent variable: maximal pain between the fibromyalgia with obsessive compulsive symptoms (OCS) group and the fibromyalgia without obsessive compulsive symptoms (OCS) group. B = beta error margin, SE = standard error, Sig = significance, * for *p*-value < 0.05.

Independent Variables		Standardized Coefficients		Sig
		B	SE	
**Maximal pain fibromyalgia without OCS**	Pain Catastrophizing—Total	0.055	0.077	0.485
	Pain Catastrophizing—Rumination	−0.034	0.027	0.230
	Pain Catastrophizing—Magnification	−0.006	0.026	0.828
	Pain Catastrophizing—Helplessness	−0.003	0.046	0.944
**Maximal pain fibromyalgia + OCS**	Pain Catastrophizing—Total *	−0.137	0.052	0.021
	Pain Catastrophizing—Rumination *	0.066	0.021	0.007
	Pain Catastrophizing—Magnification *	0.040	0.017	0.035
	Pain Catastrophizing—Helplessness *	0.097	0.034	0.012

The *p*-values presented in the table indicate the statistical significance of each independent variable in predicting the dependent variable (fibromyalgia with or without OCS). Specifically, a *p*-value less than 0.05 suggests that the corresponding independent variable significantly contributes to the prediction of the dependent variable at the 0.05 significance level. The beta coefficient (β) represents the magnitude and direction of the association between an independent variable and the dependent variable.

**Table 5:** Linear regression—pain obsessive thoughts questionnaire, dependent variable: maximal pain, average pain, B = beta error margin, SE = standard error, Sig. = statistical significance, * *p*-value < 0.05.

Dependent Variable	Independent Variables	Standardized Coefficients		Sig.
		B	Std. Error	
**Maximal pain**	Pain thought duration	−0.134	0.194	0.494
	Functional impairment *	0.535	0.207	0.015
	Anxiety	−0.099	0.201	0.625
	Pain thoughts controlling	0.457	0.227	0.053
**Average pain**	Pain thought duration	−0.269	0.161	0.106
	Functional impairment*	0.655	0.172	0.001
	Anxiety	−0.290	0.168	0.094
	Pain thoughts controlling*	0.533	0.189	0.008

The *p*-values presented in the table indicate the statistical significance of each independent variable in predicting the dependent variable (maximal and average pain). Specifically, a *p*-value less than 0.05 suggests that the corresponding independent variable significantly contributes to the prediction of the dependent variable at the 0.05 significance level. The beta coefficient (β) represents the magnitude and direction of the association between an independent variable and the dependent variable.

## Data Availability

The data presented in this study are available on request from the corresponding author. The data are not publicly available due to privacy.
